# Examining Social Capital, Social Support, and Language Use in an Online Depression Forum: Social Network and Content Analysis

**DOI:** 10.2196/17365

**Published:** 2020-06-24

**Authors:** Wenjing Pan, Bo Feng, Cuihua Shen

**Affiliations:** 1 School of Journalism and Communication Renmin University of China Beijing China; 2 Department of Communication University of California, Davis Davis, CA United States

**Keywords:** social capital, social support, social network analysis, computerized text analysis, communication accommodation, language style matching, online support forums, depression, mental health

## Abstract

**Background:**

The use of peer-to-peer online support groups and communities has grown into a social phenomenon. Many people use online support groups and communities to seek and provide social support. It is essential to examine how users’ participation behaviors may contribute to different outcomes.

**Objective:**

This study aimed to (1) use the structural positions of online depression forum users in their reply network to predict received support and (2) examine their language use reflecting their health conditions.

**Methods:**

A total of 2061 users and their 62,274 replies posted on a depression forum from July 2004 to July 2014 were extracted using a web crawler written in Python. The content of the forum users’ posts and replies and their reply patterns were examined. A social network analysis method was used to build the reply networks of users. The computerized text analysis method was used to measure features of the forum users’ language styles.

**Results:**

Forum users’ bridging social capital (operationalized as network betweenness) was positively associated with the level of communication accommodation in their received replies (*P*=.04). Forum users’ bonding social capital (operationalized as network constraint) was negatively associated with the level of communication accommodation in their received replies (*P*<.001). The forum users’ change in their use of self-referent words and words expressing negative emotions were examined as linguistic proxies for their health conditions and mental states. The results revealed a general negative association between the number of received replies and the degree of decrease in the use of words expressing negative emotion (*P*=.007).

**Conclusions:**

The structural positions of online depression forum users in the reply network are associated with different participation outcomes in the users. Thus, receiving replies can be beneficial to online depression forum users.

## Introduction

### Background

Social capital, broadly defined as the potential benefits one receives from relationships with others, is a well-researched concept in many social science disciplines. Social capital has been associated with various positive outcomes at both the individual level (such as an increased sense of trust and belonging and improved mental and physical health) [1] and societal level (such as improved public health, promoted social integration, and decreased crime rate) [2]. With the popularity of internet technologies and social networking sites, a growing number of people are turning to web-based venues to seek social support. Many studies have documented that social media and online support groups generate social capital [3].

Social support is a concept closely related to the concept of social capital. Social support encompasses the comfort, assistance, and reassurance that people experience as a function of social relationships [4]. Social support can be viewed as a product resulting from the interpersonal relationships that constitute a support network. A unique feature of seeking and providing social support online is that individuals have more opportunities to communicate with others who share similar experiences and greater access to weak-tie networks [5]. The social capital and weak-tie network theories have also been adopted to explain why and how individuals may benefit differently from participating in online support groups [6,7].

### Objectives

Previous studies investigating the network features of support seeking and provision in online support groups rarely measured the actual tie strength or examined the structural features of the support network. Furthermore, studies focusing on the content and qualities of support-seeking and support-provision messages are rare. Without examining the content of the exchanged messages, we cannot tell how support-seeking and support-provision messages are qualitatively different from each other. Utilizing big data–powered techniques such as web scraping and computerized text analysis, this study aimed to analyze the reply patterns and language styles of online depression forum users. The social capital and communication accommodation theories were adopted to make and test predictions about the association between social capital and social support of online support forum users. Drawing on the buffering effect model and self-awareness theory of depression, this study also examines the health benefits resulting from receiving social support by monitoring the changes in depression forum users’ language use over time.

### Theories and Hypotheses

#### Social Capital in Online Support Forums

**Figure 1:**
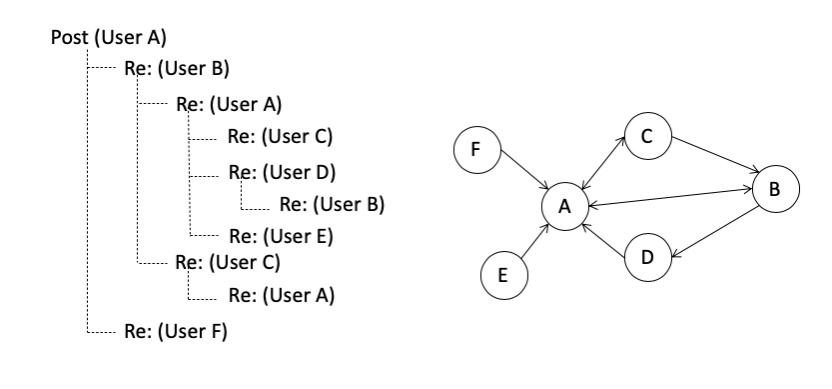
Illustration of a hypothetical user discussion network.

Active participation in an online support forum generally takes one or both of the following 2 forms: contributing an original post and responding to posts by other users. Original posts typically reflect support-seeking efforts, whereas replying to the posts of other users can be seen as support-provision efforts [7]. Replies to an original post indicate attention, engagement, and responsiveness to the posts [8,9]. Therefore, an online support forum can be conceptualized as a network of users connected by their reply-based relationships (Figure 1). An online support forum provides social capital in that it is an embedded community activated for purposeful action [10]. Social support can be viewed as an outcome of social capital because the latter provides individuals with connections or relations to receive social support from network members when needed [11].

#### Bridging and Bonding Social Capital

Social capital can be categorized into two types: bridging and bonding social capital [12]. Bridging social capital is linked to weak ties, which are loose connections between individuals and are better for linkage to external assets and information diffusion [13]. Research has shown that bridging social capital may translate to job-related information [13], higher salaries and promotion opportunities [14], and better task performance in virtual worlds [15]. Bonding social capital is inward looking and tends to reinforce exclusive identities and homogeneous groups [12]. Bonding social capital has been shown to be associated with greater emotional support, better general health and well-being, and a greater level of social trust [16].

#### Communication Accommodation Reflecting Social Support

As human beings, we consciously and unconsciously match each other’s communication acts (both verbally and nonverbally) [17]. From the message production end, communication accommodation is a socially motivated process where people engage in more or less accommodation based on their social assessment and social status of themselves and others [18]. From the message reception end, communication accommodation tends to be positively evaluated and can result in positive future intentions toward interactions with another person [18].

Individuals go to online support forums to seek and provide informational, emotional, and esteem support to each other. As these online forums are text based, various forms of support are sought and provided through text form. Communication accommodation reflected in the form of linguistic accommodation conveys a sense of caring and engagement. Linguistic accommodation can be captured through language style matching (LSM). LSM measures linguistic accommodation by taking into account the production of identical words and grammatical structures between the interactants and encompasses conversation level and synchrony on a turn-by-turn level [17]. Past research also shows that LSM among health bloggers and their readers contributes to the bloggers’ perceptions of the availability of support from the readers [19]. In this study, the level of linguistic accommodation in the received replies was employed as a proxy for social support.

#### Social Capital and Communication Accommodation

The concept of social capital can be understood structurally in that social capital refers to the “extent or the intensity of associational links or activity” [16]. The structural perspective represents an objective view of the concept of social capital, and the tie strength or the structural positions can be assessed accurately using the social network analysis method. The nature of social capital entails that, social capital, as a form of capital, can be viewed as an investment in social relations with expected returns. Bridging and bonding social capital represents two unique ways individuals choose to invest in their social relations. The different positions forum users have in the reply network represent their structural aspects of social capital. Users who possess more bridging or bonding social capital are those who (1) occupy important positions in the users’ reply social network and (2) share distinctive interaction patterns with other users. In online support forums, by investing their limited time and resources to manage their social relations, forum users expect returns in the form of social support. On the basis of the previous discussion, communication accommodation reflected in forum users’ replies can serve as an indicator of social support. In online support forums, the bridging and bonding capital of users should be positively associated with received social support. The accumulated interaction patterns among users should be associated with social support reflected in other users’ communication accommodation toward them. Therefore, based on discussion regarding the association between social capital, social support, and communication accommodation, the following hypotheses were proposed:

Hypothesis 1: Forum users’ bridging social capital will be positively associated with the communication accommodation of others in their replies.

Hypothesis 2: Forum users’ bonding social capital will be positively associated with the communication accommodation of others in their replies.

Owing to the weak nature of ties where bridging capital is accumulated, substantial emotional support or tangible support is less frequently observed compared with informational support [20]. As bonding social capital facilitates the exchange of emotional support, whereas bridging social capital facilitates the exchange of informational support, users will be more engaged in communicating with others whom they share strong ties with. The increased engagement between two communication partners also increases their degree of communication accommodation [21]. The association between users’ bridging social capital and the degree of communication accommodation in received replies should be weaker compared with the association between users’ bonding social capital and the degree of communication accommodation in received replies. Therefore, the following hypothesis was proposed:

Hypothesis 3: The association between forum users’ bridging social capital and the level of communication accommodation in their received replies will be weaker compared with the association between forum users’ bonding social capital and the level of communication accommodation in their received replies.

#### Health Outcomes of Receiving Replies

One of the most important benefits related to participation in online support groups is the improvement of the individuals’ psychological and physical health [22]. The buffering effect model maintains that social support helps individuals buffer the negative impacts caused by stressful events [23]. Drawing on the buffering effect model, studies have found that receiving social support in an online support community was negatively associated with perceived stress [24] and positively associated with online network size and perceived social support [25]. Social support exchanged in online support groups also facilitates the management of health-related uncertainty [26] and promotes positive health outcomes [27]. 

However, previous studies examining health outcomes mostly adopted self-reported measures [25,28]. Self-reported data are usually accompanied by the inaccuracy of memory or recall. Examining the changes in the language use of online support forum users can avoid (1) sampling error by web scraping and analyzing all messages exchanged in online depression forums and (2) other limitations usually associated with survey methodologies, including interviewing effect and inaccuracy of memory or recall. Several linguistic features can serve as indicators of people’s mental states [29]. Depressed individuals are more self-focused, express more negative emotions, and sometimes use more death-related words [30,31]. Research has shown that depressed patients tend to use more first-person singular pronouns and words expressing negative emotions than people who have never been depressed [32].

On the basis of previous theories regarding the buffering effect model and health benefit, it can be predicted that there should be a positive association between forum users’ number of received replies and their health improvement as reflected in their language use. Therefore, we predict the following two hypotheses:

Hypothesis 4: Forum users’ number of received replies will be negatively associated with the change in their use of first-person singular pronouns.

Hypothesis 5: Forum users’ number of received replies will be negatively associated with the change in their use of words expressing negative emotion.

## Methods

### Data Collection

**Figure 2:**
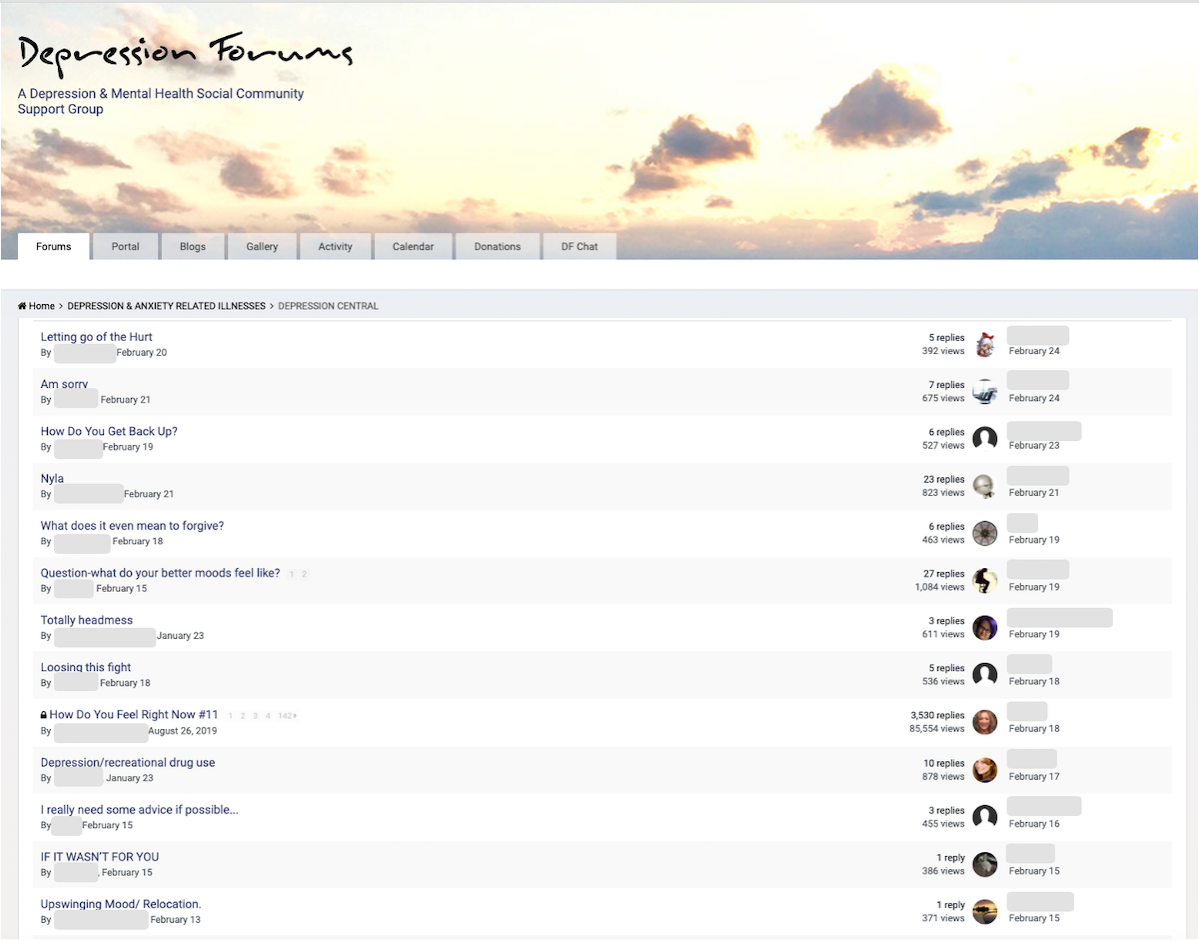
Screenshot of the depression forum’s threads. The virtual identities of the forum users are masked.

**Figure 3:**
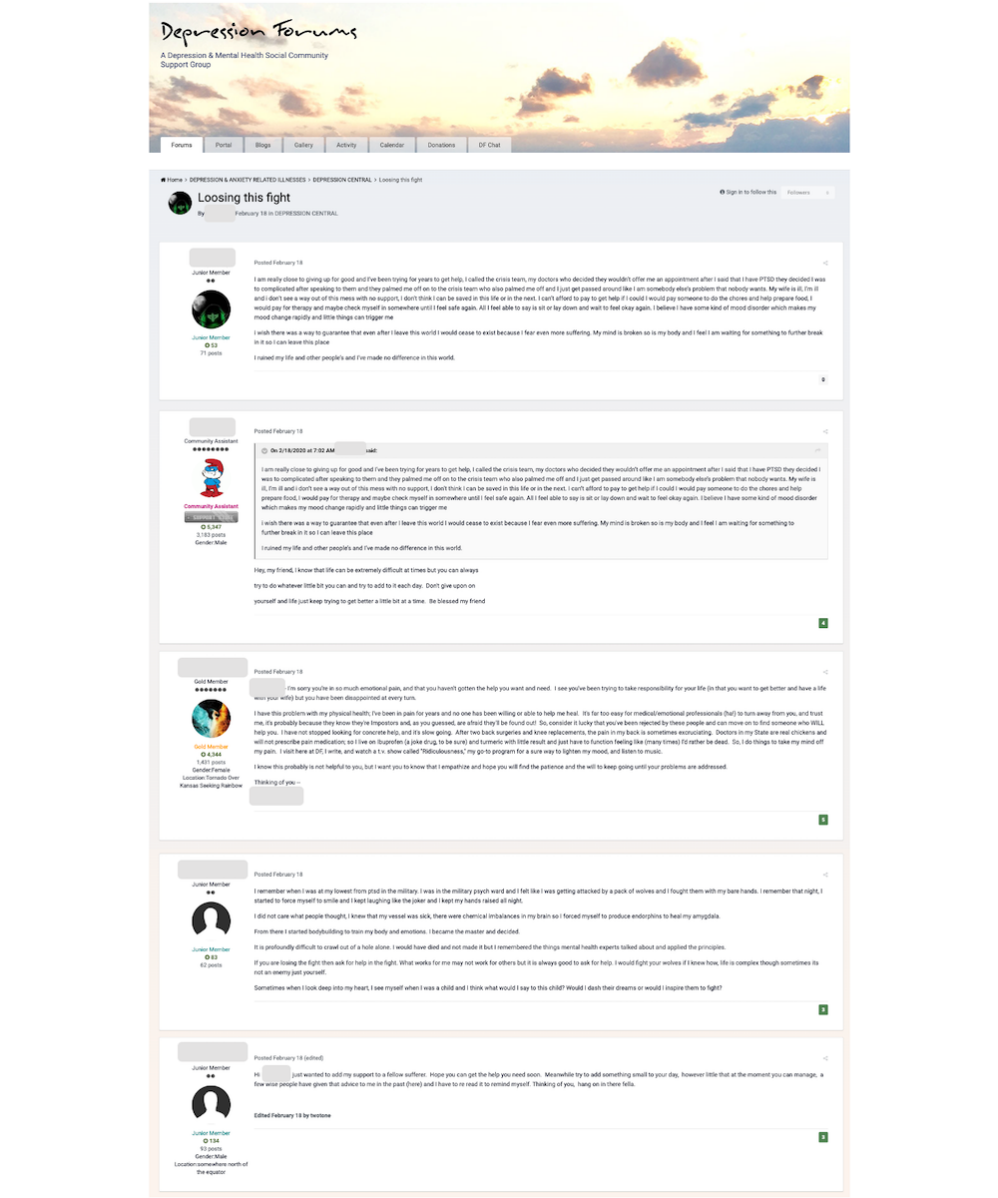
Screenshot of the depression forum’s posts and replies. The virtual identities of the forum users are masked.

The depression forum examined in this study [33] is one of the largest peer-to-peer mental health community and support group in North America (see Figures 2 and 3 for screenshots). The forum is administered and moderated by volunteers rather than health professionals. Anyone who registers as a user of the forum can make a post or reply to others to participate in forum discussion.

Web-scraped data from the depression forum were used for this study [6]. The original forum was written in hypertext markup language in a structured format. The web crawler was written in the computer programing language, Python. Forum data including the title of the subforums, the title of the thread, post ID, reply ID, time stamps, full texts of the posts, and replies were extracted. The web-scraped data were then converted into JavaScript Object Notation (JSON) files. To perform a social network analysis, the adjacency matrix in comma separated value (csv) was constructed from the JSON file using the Python code. The Python code for the web crawler and network data extraction can be found in Multimedia Appendices 1 and 2.

The complete data set included 34,554 users and their 63,514 posts and 592,649 replies from July 2004 to July 2014. Users who never made any posts or replies (n=17,572) were removed from the analysis. The mean number of sent-out replies was 10 (SD 74.23), and the mean for the number of received replies was also 10 (SD 36.05). Furthermore, more than 94.04% (32,493/34,553) of users had <10 sent-out and received replies combined over 10 years. Therefore, following a procedure similar to that of previous studies to capture users’ active participation, only users who contributed ≥10 sent-out and received replies in total were included in the final data set [34,35]. The final data set included 2061 users and their 62,274 replies".

### Data Analysis

On the basis of the final data set, an adjacency matrix was created to represent the users’ reply patterns, with rows and columns representing each user and the number in each cell representing the number of replies between any given 2 users. Packages *sna* [36] and *network* [37] in the R programing language were used to calculate network-related variables such as the number of received replies, number of sent-out replies, betweenness, and constraint.

To calculate the forum users’ language use styles, a computerized text analysis program, linguistic inquiry and word count (LIWC; Pennebaker, Booth, and Francis), was used [38]. LIWC is a dictionary-based software that compares and matches textual messages with predefined word categories. LIWC has been widely adopted and validated to examine the content and style features of various texts [30]. IBM’s SPSS version 26 was used to perform linear regression analyses.

### Measures

#### Bridging Social Capital

Following similar studies of online social capital [39], bridging social capital was structurally measured by each node’s network betweenness. Betweenness measures the extent to which the actor falls on the geodesic (shortest) paths between other pairs of actors in the network [40]. Definitions of the key variables, operationalization, and measurements are included in Table 1.

**Table 1:** Definitions, operationalizations, and measurements of the key theoretical constructs.

Concepts	Definitions	Operationalizations	Measure
Bridging social capital	Bridging social capital is linked to weak ties, which are loose connections between individuals and are better for linkage to external assets and information diffusion	Network structural representation of structural hole or brokerage	Betweenness: Suppose that *g*_i_^(^^st^^)^ is the number of geodesic paths from actor *s* to actor *t* that pass through *I*, and that *n*_st_ is the total number of geodesic paths from *s* to *t.* The betweenness of the actor *i* can be given in the following formula [41]: 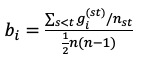
Bonding social capital	Bonding social capital is inward looking and tend to reinforce exclusive identities and homogeneous groups	Network structural representation of closely connected components	Constraint [42]: 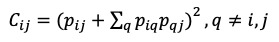 where *p*_ij_ is the proportion of ego *i*’s resource invested in connection with *j* and *p*_qj_ is the strength of *q*’s tie to *j*.
Social support	Social support encompasses the comfort, assistance, and reassurance that people experience as a function of social relationships	Communication accommodation reflected in forum users’ posts and replies	Language style matching (LSM): For example, for personal pronouns (pp) between a given post of user A and the first received replies, the calculation would be: 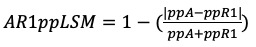
Health conditions	Language use can reflect individuals’ psychological states and health conditions	(1) Use of self-referent words (2) Use of words expressing negative emotion	(1) Change in the use of self-referent words=the number of self-referent words in last 10 replies−the number of self-referent words in the first 10 replies (2) Change in the use of words expressing negative emotion=the number of words expressing negative emotion in last 10 replies−the number of words expressing negative emotion in the first 10 replies

#### Bonding Social Capital

Bonding social capital was measured structurally as a network constraint. Constraint is “a concentration measure that varies from zero to 100 with the extent to which all of a person’s network time and energy is concentrated in one contact” [42]. A large number of constraints suggest that the ego invests most resources within a small and connected group of nodes.

#### Language Style Matching

LSM scores were calculated for each user to measure the extent to which others have used similar language styles [19,43,44]. LSM was operationalized as the degree to which a user’s post and received replies from others used a similar proportion of 9 classes of function words. Each forum user’s use of 9 types of function words in each of their posts was calculated. The 9 types include auxiliary verbs (eg, to be, to have), articles (eg, an, the), common adverbs (eg, hardly, often), personal pronouns (eg, I, they, we), indefinite pronouns (eg, it, those), prepositions (eg, for, after, with), negations (eg, not, never), conjunctions (eg, and, but), and quantifiers (eg, many, few). To capture the nuance of language use similarity within each thread, the percentage of total words for each of the 9 types of function words was then calculated for each post and subsequent replies. On the dyadic level, between each user’s original posts and each of his or her received replies, the absolute value of the difference was divided by the total for each category. The LSM score can only range from 0 to 1, with scores closest to 1 reflecting high degrees of style matching [43]. The formula used to calculate the LSM is shown in Table 1.

For example, user A made a post and received 3 replies. The personal pronoun LSM for user A’s first post was calculated for each of the 3 replies, AR1ppLSM, AR2ppLSM, and AR3ppLSM. Then, the overall personal pronouns LSM score for user A’s first thread was calculated by taking the average of the personal pronouns LSM between user A and all of the received replies under the same thread. In the case of user A receiving 3 replies, the overall personal pronoun LSM score for user A is calculated as the mean of the 3 personal pronoun LSM scores.

As there are 9 separate dimensions of function words that make up the overall LSM score, similar calculations were conducted for each user’s thread for each function word category. For each user’s thread, the 9 separate mean LSM scores for each category were averaged to yield a total LSM score. The same process was applied to each user’s thread. The final LSM score for a given user was calculated by taking the average of all threads.

#### Number of Received Replies

The number of received replies of the forum users were operationalized as the total number of replies one user received from others.

#### Change in Language Use

In this study, linguistic markers including first-person singular pronouns and words expressing negative emotion were examined. First-person singular pronouns include pronouns such as *I*, *me*, *mine*, and *my* and negative emotions words include words regarding anxiety, anger, and sadness (eg, *hate, worthless, afraid, cry*). For change in first-person singular pronouns, the percentage of first-person singular pronouns in their first 10 messages was subtracted from the percentage of first-person singular pronouns in their last 10 messages. The same process was applied to the calculation of the change in words expressing negative emotion.

## Results

### Preliminary Analysis Results

**Figure 4:**
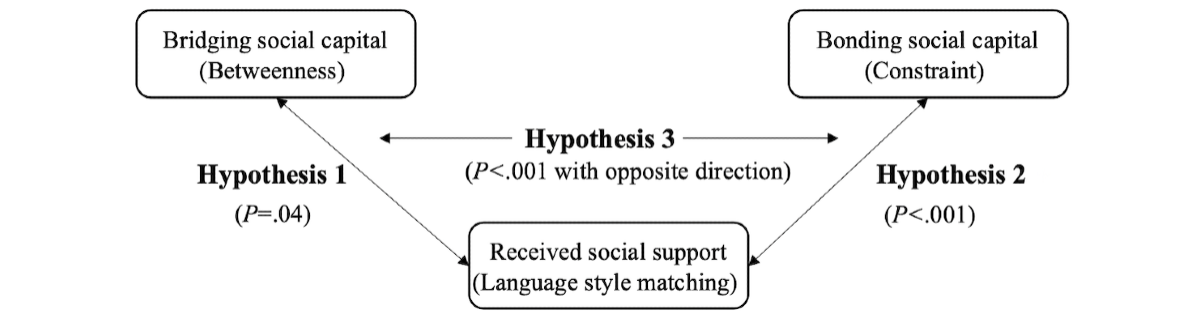
Theoretical framework and results for Hypothesis 1, Hypothesis 2, and Hypothesis 3.

**Figure 5:**
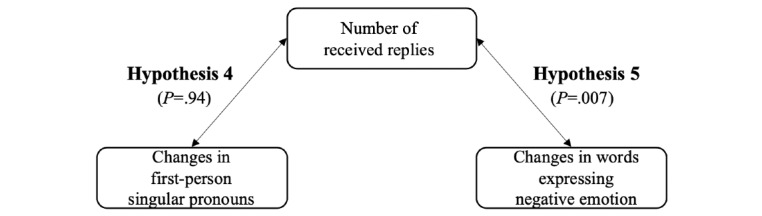
Theoretical framework and results for Hypothesis 4 and Hypothesis 5.

The final forum user reply network included 2061 users, and 62,274 replies. On average, each user received 29.23 (SD 44.09) replies and sent out 29.22 (SD 58.20) replies to others. Each user, on average, engaged in interaction with 11 other users (SD 5.00). The average length of their received replies was 155.85 (SD 144.00) words. Descriptive statistics of the key variables are included in Table 2. Theoretical models and results of the hypotheses testing are shown in Figures 4 and 5.

**Table 2:** Descriptive statistics and zero-order correlation among variables.

Variables		Betweenness	Constraint	Received replies	LSM^a^	Change in first-person singular pronouns	Values, mean (SD)
**Betweenness**							0.77 (7.57)
	*r*	—^b^	—	—	—	—	
	*P* value	—	—	—	—	—	
**Constraint**							0.05 (0.03)
	*r*	−0.14	—	—	—	—	
	*P* value	<.01	—	—	—	—	
**Received replies**							29.23 (44.09)
	*r*	0.59	−0.46	—	—	—	
	*P* value	<.01	<.01	—	—	—	
**LSM**							0.21 (0.10)
	*r*	0.08	−0.13	0.20	—	—	
	*P* value	<.01	<.01	<.01	—	—	
**Change in first-person singular pronouns**							0.01 (2.29)
	*r*	−0.01	−0.03	0.01	0.01	—	
	*P* value	—	—	—	—	—	
**Change in words expressing negative emotion**							0.17 (1.38)
	*r*	−0.10	−0.05	−0.05	−0.01	0.15	
	*P* value	<.01	<.05	<.05	—	<.01	

^a^LSM: language style matching.

^b^Not applicable.

### Hypotheses Testing Results

H1 predicted a positive association between users’ bridging social capital (betweenness) and their overall LSM. As the LSM score is likely to be affected by the number of users one receives replies from, it was then included in the regression model as a control variable. The analysis revealed that, after controlling for the number of users one received replies from, betweenness (mean 0.77, SD 7.57) was positively associated with the overall LSM (mean 0.21, SD 0.10; *β*=.06; t_2059_=2.05; *P*=.04). Bridging social capital explained a small but significant portion of the variance in users’ overall LSM (*R^2^*=0.03; *F*_1,2059_=43.84; *P*=.001). Therefore, H1 was supported.

H2 predicted a positive association between users’ bonding social capital (constraint) and their overall LSM. After controlling for the number of users, one received replies from constraint (mean 0.05, SD 0.03) was negatively associated with the overall LSM (mean 0.21, SD 0.10; *β*=−.17, t_2059_=−6.72; *P*<.001). Bonding social capital explained a significant portion of the variance in users’ overall LSM (*R^2^*=0.04; *F*_1,2059_=63.24; *P*<.001). This finding was not consistent with the hypothesis. Therefore, H2 was not supported.

H3 predicted that the association between forum users’ bonding social capital and LSM would be stronger compared with the association between forum users’ bridging social capital and LSM. Standardized *z* scores for LSM were entered as dependent variables, whereas standardized betweenness and constraint scores were entered as independent variables [45]. The corresponding 95% CIs were estimated via bias-corrected bootstrapping (1000 resampled). The CI for betweenness was 0.04 to 0.38, whereas the CI for constraint was −0.19 to −0.05. Therefore, the proportion overlap between the 2 CIs was negative (*P*<.001). This result showed that the association between bonding social capital and LSM was stronger than the association between bridging social capital and LSM. However, the direction of the association between bonding social capital and LSM was the opposite of the prediction. Therefore, H3 was not supported.

H4 predicted a positive association between the users’ received replies and their use of first-person singular pronouns change. As the users’ language use change is likely to be affected by the number of replies they sent out, it was then included in the regression model as a control variable. Considering that the change in each user’s use of first-person singular pronouns was calculated based on the differences between their first and last 10 messages, each user’s length of forum use on the forum could also affect their change in language style. Therefore, each user’s length of forum use was included in the regression model as a control variable. After controlling for the number of sent-out replies and length of forum use, regression analyses showed that the number of received replies (mean 29.23, SD 44.09) was not significantly associated with the change in their use of first-person singular pronouns (mean 0.01, SD 2.29; *β*=.01; t_2059_=0.07; *P*=.94). Therefore, H4 was not supported.

H5 predicted a negative association between the users’ number of received replies and their use of words expressing negative emotions changed over time. Similar to the analysis of H4, each user’s number of sent-out replies and length of forum use were also included in the regression model as control variables. After controlling for the number of sent-out replies and length of forum use, regression analyses showed that users’ number of received replies (mean 29.23, SD 44.09) was negatively associated with the change in their use of words expressing negative emotions changed over time (mean 0.17, SD 1.38; *β*=−.06; t_2059_=−2.69; *P*=.007). The users’ number of received replies explained a significant portion of the variance in users’ use of words expressing negative emotions changed over time (*R^2^*=0.004; *F*_1,2059_=4.39; *P*=.01). H5 was supported.

## Discussion

### Principal Findings

Depression, as a common mental disorder, has been estimated to affect more than 264 million people worldwide [46]. Owing to its detrimental effects on the individual’s psychological and physical health, scholars and practitioners are dedicated to the prevention, diagnosis, and treatment of depression. Other than seeking help from professionals, individuals with depressive symptoms sometimes turn to online support groups and forums to seek, provide, and exchange social support. Online support groups and forums provide them with access to others who share similar experiences at a low cost. Therefore, studying peer-to-peer support exchanged in these online support groups and forums posits both practical and theoretical importance.

Adopting conceptualizations of social capital and its 2 forms, this study differentiated between 2 distinctive patterns (bridging reflected in betweenness and bonding reflected in constraint) of participation behaviors of online forum users. This study also investigated the associations between online support forum users’ social capital and social support in the form of linguistic accommodation. Furthermore, this study also examined the association between receiving replies and health conditions as reflected in forum users’ language use. The findings and their implications are discussed in the following paragraphs.

First, the associations between forum users’ social capital and their received support were examined. Using social network structural measurement of social capital based on the users’ reply patterns, this study found that forum users’ bridging social capital was positively associated with the received support, as reflected in the level of linguistic accommodation. This result is in line with previous research examining the association between the positions of online network members and their influences on other networks [9] as well as the association between network positions and their perceived or received support from the network [6]. Network members who possess more central positions tend to stay active in health communities and are more likely to share informational support with less central members [47]. When online forum users engage in interactions with other members, they invest their limited time and attention. From the cost-benefit perspective of social capital, this invested social capital in the form of attending to the support-seeking efforts of others can bring them benefits. For example, the users’ bridging capital has been positively associated with the diversity of received replies, whereas users’ bonding capital has been positively associated with the length of received replies [6]. Similarly, this study also found that users serve as bridges that connect otherwise unconnected others together may also receive a higher level of linguistic accommodation from others.

However, in terms of the association between users’ bonding social capital and received replies, the result indicated an opposite pattern. Forum users’ bonding social capital was negatively associated with the level of linguistic accommodation in the received replies. Communication accommodation is a complex phenomenon that can be promoted by various perceptions and social processes. One line of research sees communication accommodation as a reflection of mutual trust, liking, or rapport [48], whereas another line of research treats communication accommodation as being promoted by social perceptions reflecting engagement as well as expertise judgment [49]. Predictions rooted in the rapport perspective would support a positive association between LSM and bonding social capital because the latter facilitates the building of trust and emotional bonds among members, whereas researchers focusing on the engagement perspective see LSM as a reflection of communication partners’ involvement and engagement. For example, the use of words expressing emotion from posters can elicit a higher level of engagement and involvement from others [44].

Additional analysis showed that the bonding capital of the forum users was negatively associated with their use of words expressing emotion (*β*=−.05; t_2059_=−2.34; *P*=.02). In other words, the more users possessed bonding social capital, the less they will use emotional words in their posts. The less they used emotional words in their posts; the less others were mimicking their language style when replying. The findings on the negative association between bonding social capital and LSM suggest that the underlying mechanism of LSM could be multifaceted, and accommodation could be a function of many social processes such as engagement rather than building rapport or liking.

Expertise judgment, as outlined in expectation states theory (EST), can also shed light on unexpected findings in which a negative association between bonding social capital and LSM was observed [50]. According to EST, individuals use characteristics indicating pre-existing status (eg, gender, race) and behavioral interchange patterns within-group processes to judge the competence of others. Characteristics indicating pre-existing status can be directly communicated or inferred through speech and nonverbal cues, whereas interchange patterns can be observed during interactions with others [49]. In this study, the language used by a given user and the linguistic accommodation of others can serve as characteristics indicating pre-existing status and behavioral interchange patterns, respectively. For users who possess more bonding social capital, their language style is less affective and less accommodated by others. They can both serve as grounds for others to form expert judgments. Therefore, users who possess more bonding capital are likely to be viewed as possessing less expertise and a low social status. Thus, others are less likely to accommodate their language style. Although without further examination of the users’ motives or subjective experience of their interactions with other forum users, it remains an empirical question as to what cues forum users utilize to form judgments or perceptions about other users. However, in text-based online support forums with a limited number of cue systems, the language use of forum users can nonetheless serve as an important ground for users to form perceptions about each other.

One caveat regarding the association between the users’ social capital and LSM in their received replies lies in the direction of the association. Social capital and social support theories may evaluate LSM as an outcome or reflection of interpersonal and social processes, whereas virtual team dynamics and related computer-mediated communication (CMC) theories may see network positions and formations as outcomes of LSM because of the text-based nature of CMC. For example, language features of the user him or herself and the perceived LSM of the user can both be associated with the user’s network position. As the LSM was calculated between a user’s original post and the received replies to the same post, language features of the poster could affect the level of linguistic adaptation from others. The forum user’s own language features can also be affected by their network positions or the composition of their social networks. For example, the heterogeneity of the social networks of Facebook users is negatively associated with language style variability, as reflected in their status updates [34]. The lowest common denominator approach to online self-presentation posits that individuals will choose to share information that is considered appropriate for all audience segments [51]. The proposed pattern has been observed in the status updates of Facebook users: users tend to vary little in their language style when the audience segments are disconnected with each other [34]. Online support forum users who possess more bridging social capital (higher network betweenness) share ties with heterogeneous groups of others. On the basis of the lowest common denominator approach, these users are assumed to have less variability in their language style. Therefore, the replies they received from others share more similarities with their own language style, given that a user’s LSM is calculated between each post contributed by the same user and each reply addressing the same post.

The results of this study showed a positive association between the number of received replies and a decrease in the use of words expressing negative emotion. These results are in line with the self-awareness theory of depression [52] and previous literature on language features of depressed people. As discussed earlier, words expressing negative emotion are important linguistic markers for identifying depression. Depressed individuals are more self-focused and express more negative emotions [30]. Previous studies have established that language features can be markers of mental health [32]. The results suggested that for depression forum users, after controlling for their length of forum use, receiving replies was associated with improvement in their psychological states, although causality could not be established because of the nature of the data. Further mediation analysis showed that the level of communication accommodation exemplified in the users’ received replies did not mediate the effects of bridging and bonding social capital on the users’ change in their use of self-referent words or words expressing negative emotion (Multimedia Appendix 3 shows mediation results).

### Limitations

Several limitations of the study are worth noting. The first limitation lies in the lack of causality in the findings. As the users’ bridging and bonding social capital, as well as the LSM, was treated cross-sectional as individual attributes, causal relations cannot be established. Although the changes in the language features of forum users can serve as longitudinal indicators of their language use tendency, the data were treated as cross-sectional. Although the benefit of web-scraped forum users’ participation patterns is to offer an unobtrusive way to collect the users’ actual behavioral data, future studies could split data into different periods and observe the trend in the users’ social capital and language use to establish causal relations between forum participation and related outcome measurements.

The second limitation lies in the lack of content measures for exchanged messages. Although computerized text analyses offer powerful and effective ways to investigate the forum users’ language features, the context is largely ignored. Due to the exclusive reliance on words recorded in the analytical software’s dictionaries, the assessment of the outcomes of interest can be somewhat rigid. Future research could use human coders to cross-validate computerized text analysis results.

### Conclusions

This study contributes to our understanding of how people interact with each other in online support forums. In particular, we extend the research focus of online supportive communication to the network positions of users and the communication accommodation of their received support. Using a social network analysis method to analyze web-scraped data from an online support forum, this study offers an objective and nuanced way to examine the structural aspect of social capital. Analyzing the linguistic features of messaged exchanged in online support forums, this study also offers an unobtrusive way to analyze the actual messages created and used by online support forum users.

## Appendix

Multimedia Appendix 1 Python code for web crawler.

Multimedia Appendix 2 Python code to process network data.

Multimedia Appendix 3 Mediation results of communication accommodation mediating the effects of 2 types of social capital on users’ change in self-referent words and words expressing negative emotion.

## Abbreviations

CMC: computer-mediated communication
EST: expectation states theory
JSON: JavaScript Object Notation
LIWC: Linguistic Inquiry and Word Count
