# Supplementary material for: Examining Social Capital, Social Support, and Language Use in an Online Depression Forum: Social Network and Content Analysis
Source: J Med Internet Res. 2020 Jun 24;22(6):e17365. doi: 10.2196/17365 (PMC7381041; doi:10.2196/17365)
Supplement: Multimedia Appendix 3 [file jmir_v22i6e17365_app3.docx]

Follow-up mediation analyses were conducted to examine if communication accommodation (LSM) mediated the effects of two types of social capital (as reflected in users’ network structural positions) on psychological outcomes manifested in language use (change in the use of first-person singular pronouns and change in the use of negative emotion words).

In order to test for the mediation effect, PROCESS macro developed by Andrew Hayes (2017) in SPSS was adopted. The indirect effect of betweenness on change in the use of first person-singular pronouns mediated through LSM was not significant (*b* = .16, *SE* = 1.38, 95% CI = [−2.6217, 3.8478]) after bootstrapping with 5,000 resamples. The indirect effect of betweenness on change in the use of negative emotion words mediated through LSM was not significant (*b* = -.03, *SE* = .78, 95% CI = [−2.0768, 1.4225]) after bootstrapping with 5,000 resamples.

The indirect effect of constraint on change in the use of first person-singular pronouns mediated through LSM was not significant (*b* = -.03, *SE* = .28, 95% CI = [− .6210, .5014]) after bootstrapping with 5,000 resamples. The indirect effect of constraint on change in the use of negative emotion words mediated through LSM was not significant (*b* = .12, *SE* = .16, 95% CI = [−.1280, .3427]) after bootstrapping with 5,000 resamples.

Therefore, communication accommodation did not mediate the effects of two types of social capital (as reflected in users’ network structural positions) on psychological outcomes manifested in language use (change in the use of first-person singular pronouns and change in the use of negative emotion words).
